# An integrated microfluidic device for screening the effective concentration of locally applied tacrolimus for peripheral nerve regeneration

**DOI:** 10.3892/etm.2014.2082

**Published:** 2014-11-19

**Authors:** BAO-SHENG YIN, MING LI, BO-MING LIU, SHOU-YU WANG, WEI-GUO ZHANG

**Affiliations:** Department of Orthopedics, The First Affiliated Hospital of Dalian Medical University, Dalian, Liaoning 116011, P.R. China

**Keywords:** microfluidic device, tacrolimus, effective concentration

## Abstract

The effectiveness of tacrolimus (FK506) for the promotion of nerve regeneration is known. However, at present, due to the fact that systemic application may lead to opportunistic infections and tumors, and that the treatment of peripheral nerve injury with systemic immunosuppression is not generally accepted, FK506 has not been widely used for the treatment of simple or peripheral nerve injury. In this study, a pyramid-shaped microfluidic device was designed and fabricated that was able to analyze the effective concentration of locally applied FK506. After testing the effectiveness of the microfluidic device by measuring the fluorescence intensity of fluorescein isothiocyanate-dextran, rat Schwann cells (SCs) were loaded into the device and cultured for 9 days in the presence of different concentrations of FK506. SC proliferation in the presence of FK506 was concentration-dependent between 0 and 2.5±0.003 ng/ml. The proliferation rate reached a maximum at 1.786±0.014 ng/ml, which was statistically significantly different from the proliferation rate at lower FK506 concentrations. There was no statistically significant difference in the proliferation rate between the 1.786 ng/ml group and groups of higher FK506 concentrations. Furthermore, the SCs in the microfluidic device and a 96-well plate continued to proliferate as the culture time increased. No statistically significant differences were identified between the microfluidic device and a 96-well plate with regard to the proliferation rates in each corresponding group. The results obtained in this study demonstrated that the microfluidic device can be used as an excellent platform for the study of drug concentration at the cellular level, and the effective FK506 concentration for local application is 1.786±0.014 ng/ml.

## Introduction

The repair of peripheral nervous system (PNS) damage resulting from trauma, excessive stretching and even iatrogenic injury is a tremendous challenge for clinical medicine as the recovery of nerve function may be poor due to broken end distortion following nerve anastomosis and scar ingrowth, ineffective nerve regeneration and slow regeneration. Among several current therapies for PNS damage, for example, neurosuture and nerve grafting, the combined application of nerve regeneration-promoting drugs is a promising technology that has few adverse reactions (1). Tacrolimus (FK506) is a macrolide immunosuppressant approved by the US Food and Drug Administration. It plays a role not only in immunosuppression but also in the effective promotion of nerve regeneration. However, at present, the systemic application of FK506 has certain side-effects, such as nephrotoxicity, gastrointestinal dysfunction and hypertension (2–5). In current research, the local application of FK506 is regarded as a feasible method for reducing side-effects. However, no accurate test is available to evaluate the effective concentration for local application (6). A newly emerging technology known as microfluidics has been demonstrated to be very useful for screening the dosages of drugs. A stable cell culture environment can be constituted and maintained by a continuous medium supply and waste removal system that resembles the human circulatory system. Furthermore, it reduces the number of cells required and the requirement for large volumes of culture medium and costly reagents, which makes the microfluidic device an attractive platform for high-throughput screening (7,8).

In the present study, an integrated microfluidic device was designed and fabricated that may provide an ideal platform for cell level manipulation and analysis *in vitro*. Furthermore, rat Schwann cells (SCs) were loaded into the device and the optimum concentration of FK506 was determined with the aim of providing an experimental and theoretical reference for the therapy of peripheral nerve injury.

## Materials and methods

### Materials

FK506 (molecular weight, 804.02 g/mol) was purchased from Purac Biochem Co. (Gorkum, The Netherlands), and used without further purification. Rat SCs (RSC96 cell line) were purchased from ScienCell Research Laboratories (Carlsbad, CA, USA).

### Design and fabrication of the microfluidic device

**Figure 1:**
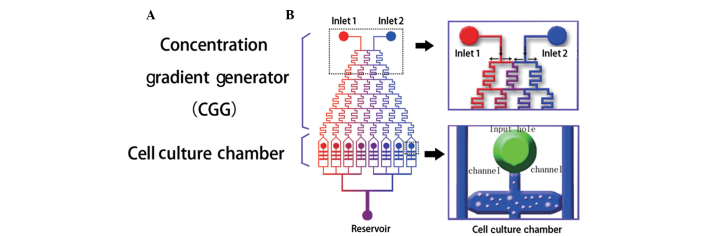
Microfluidic device design. (A) Schematic drawing of the microfluidic device that is composed of an upstream concentration gradient generator and a downstream cell culture chamber. When two solutions, represented by two colors, are injected into a device through inlet 1 and inlet 2, they are gradually diluted and form a gradient. (B) The two solutions are split at the nodes, combined with neighboring streams in a laminar fashion, and mixed by diffusion in the serpentine channels. (C) Schwann cells were loaded into the cell culture chamber through an input hole, and tacrolimus (FK506) diffused into the chambers by osmosis.

The microfluidic device was designed using computer-aided design (CAD) software, to provide a continuous concentration gradient for the chemical stimulation of a cell culture (9). The device included two parts (Fig. 1A), namely a pyramid shaped concentration gradient generator (CGG) and a cell culture chamber. In accordance with the Reynolds effect, the Reynolds number can be very low when the capillary diameter is ~100 nm to several hundred micrometers, and liquid will show a laminar flow when it passes through the serpentine channels of the microfluidic chip. When using this chip, if one solution (concentration A) and a second solution (concentration 0) are injected into the CGG via the two inlets, they are split at the nodes, combined with neighboring streams in a laminar fashion, and mixed by diffusion in the serpentine channels, and the concentrations in the eight outlets are, respectively, 0, 1/7A, 2/7A, 3/7A, 4/7A, 5/7A, 6/7A and A.

Three cell culture chambers of the same function were connected between two parallel channels of each outlet of the CGG. The volume of each chamber was 0.4 μl (length, 800 μm; width, 500 μm; height, 100 μm). The SCs were loaded into a chamber through an input hole. When solutions flow down the parallel channels, substances contained in them, such as FK506, diffuse into the chamber by osmosis until saturation is achieved.

The microfluidic devices were fabricated in poly-dimethylsiloxane (PDMS) using rapid prototyping and soft lithography (11). Firstly, a transparency mask was generated by a high resolution printer from the CAD file. The mask was used in 1:1 contact photolithography with an SU-8 photoresist (MicroChem, Newton, MA, USA) to generate a negative master consisting of a patterned photoresist on a silicon wafer. Positive replicas with embossed channels were fabricated by molding PDMS against the master. Secondly, the inlets and outlets (ϕ=1 mm) for the fluids were punched out of the PDMS using a sharpened needle. Then, an ultrasonically cleaned glass substrate and the PDMS molding were irreversibly combined together to form a system of microfluidic channels in the PDMS-glass composite chip. The channels were 100 μm wide and 30 μm deep.

### CGG performance validation

Fluorescein isothiocyanate-dextran (FITC-dextran; Nanocs, Boston, MA, USA) was used as an indicator for evaluating the gradient generated by the CGG. By injecting FITC-dextran and phosphate-buffered saline into the two inlets, respectively, the FITC-dextran was continuously diluted. A series of concentrations of FITC-dextran were thus acquired in the CGG and then flowed into the corresponding downstream cell culture devices. As the intensity of fluorescence of FITC-dextran is proportional to its concentration, the concentration was positively associated with the intensity of fluorescence of FITC-dextran in the CGG (10). The intensities of FITC-dextran at the eight outlets of the CGG were imaged by confocal laser scanning microscopy (Leica Microsystems, Wetzlar, Germany) and quantified using Image-Pro Plus software (version 6.0 for Windows 7; Media Cybernetics, Inc., Rockville, MD, USA). All experiments were repeated three times. The measured values were compared with theoretical values (9), whereafter, their relativity was analyzed.

### Screening of the effective concentration of FK506

**Figure 2:**
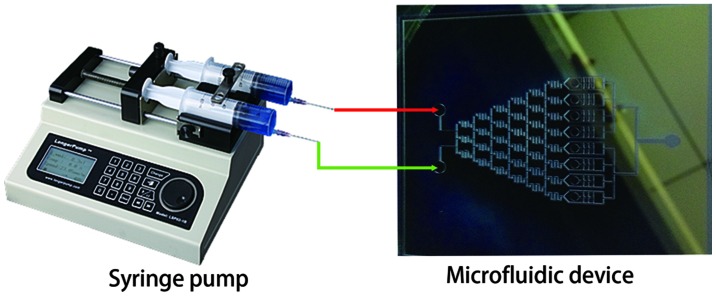
Microfluidic device. A syringe pump is connected to the microfluidic device.

Culture media with and without FK506 were simultaneously infused into the microfluidic device. The fluid was driven by a medical syringe pump, and the flow speed was controlled at 0.1 μl/min (Fig. 2). The concentration gradient of FK506 was established at the eight outlets of the CGG 30 min later following the repeated splitting, mixing, and recombination of the fluid streams as they traveled through the channels of the CGG. In each cell culture chamber to which the SCs had been loaded, a culture medium with different concentrations of FK506 was obtained when the solution flowed in the parallel channels of each outlet (12). To prevent the washing out of cells, a 3-h incubation step was conducted prior to infusion of the culture media since SCs loaded inside the chambers attach to FK506 after a while and their locations remain relatively stable. The device was then kept in an incubator at 37°C, 5% CO_2_ and 100% humidity for an additional 1, 3, 5, 7 and 9 days. At every indicated time interval, the extent of the cell proliferation was evaluated using a cell counting kit 8 (CCK-8) assay (Dojindo, Tokyo, Japan) (13). Then, the concentration of FK506 was analyzed by liquid chromatography-mass spectrometry (3200QTRAP^®^; Applied Biosystems, Foster City, CA, USA). All cell proliferation assays were repeated in 96-well plates with the same concentrations.

### Statistical analysis

All quantitative data were analyzed and expressed as the mean ± standard deviation. Cell proliferation assay results were assessed by one way analysis of variance (ANOVA). The comparison between two means was analyzed using Tukey’s test, and P<0.05 was considered to indicate a statistically significant difference.

## Results

### CGG performance validation

**Figure 3:**
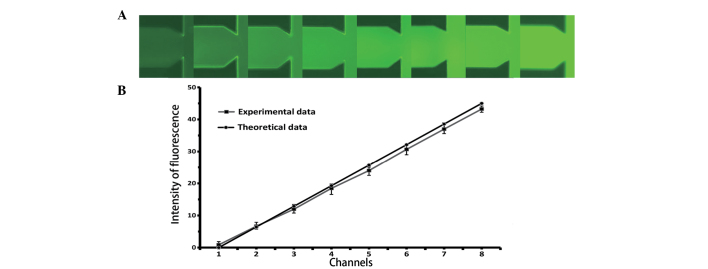
Concentration gradient generator (CGG) performance validation. (A) Images of fluorescein isothiocyanate (FITC)-dextran at downstream chambers of eight outlets of the CGG by confocal laser scanning microscopy. (B) The experimental intensities of FITC-dextran were quantified and compared with the theoretical values.

Figs. 1 and 2 are images of the microfluidic device. As streams of dye traveled down the network, they were repeatedly split, combined with neighboring streams in laminar mode at the nodes, and mix by diffusion in the serpentine channels. A series of solutions with different concentrations of FITC-dextran were formed at the cell culture chamber (Fig. 3A). The fluorescent intensities of FITC-dextran in the junctions between the CGG and the cell culture module were quantified, corrected by subtraction of the background fluorescence, and compared with the theoretical data. As shown in Fig. 3B, there was a good coherence (correlation coefficient = 0.9948) between the experimental and theoretical data.

### Cell proliferation and effective concentration of FK506

**Figure 4:**
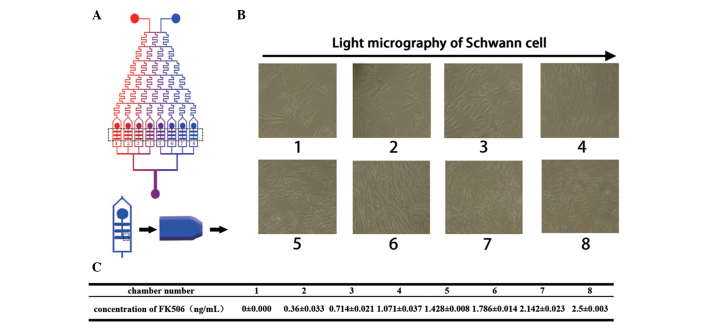
Cell proliferation in the microfluidic device. (A) Schwann cells (SCs) were loaded into the device and cultured for nine days. (B) Light micrographs taken from every third cell culture chamber of the eight groups. (C) The concentrations of FK506 at each outlet.

**Figure 5:**
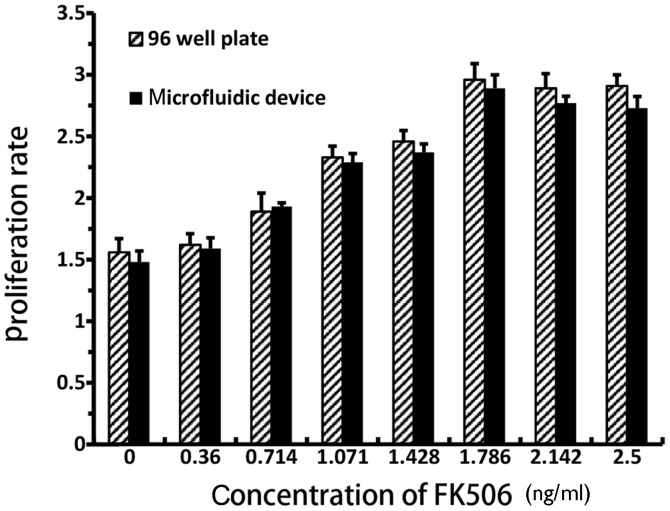
Proliferation rate of Schwann cells between 0 and 2.5 ng/ml as determined by the microfluidic device and a 96-well plate. .

**Figure 6:**
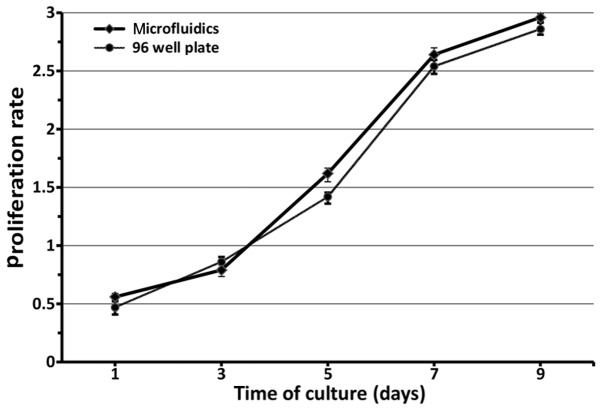
Comparison of the proliferation rate of Schwann cells between the chip and 96-well plate for an FK506 concentration of 1.786 ng/ml.

To measure the effective concentration of FK506, SCs were loaded into the microfluidic device and exhibited a polygonal morphology after nine days in the presence of different concentrations of FK506 solution (Fig. 4B). The effective concentration was screened over a large range, and it was confirmed that the highest proliferation rate was obtained at a concentration between 1.786±0.014 and 2.5±0.003 ng/ml. The concentrations of FK506 from chamber 1 to chamber 8 are shown in Fig. 4C. The concentration-dependency of SC proliferation in the presence of FK506 was apparent between 0 and 2.5±0.003 ng/ml as shown in Fig. 4B. The proliferation rate reached a maximum in the 1.786±0.014 ng/ml group (Fig. 5), which was statistically significantly different from the proliferation rates in the groups of lower FK506 concentrations (P<0.01). These results were consistent with the light micrographs shown in Fig. 4B. There was no statistically significant difference between the proliferation rate in the 1.786±0.014 ng/ml group and that in the groups of higher FK506 concentration (P>0.05). Furthermore, in Fig. 6, the proliferation of RSCs was evaluated by CCK-8 quantitative assay after culturing for nine days. The RSCs in the microfluidic device and 96-well plate continued to proliferate as the culture time was prolonged. There were no statistically significant differences between the microfluidic chips and 96-well plate with regard to proliferation rate in each corresponding group (F=121.366, P>0.05). Thus, it is considered that the effective local concentration of FK506 is in the vicinity of 1.786±0.014 ng/ml.

## Discussion

Microfluidic devices integrate the preparation of pharmaceutical compositions, separation, detection, cell culture and other basic operations into a very small chip (14). Compared with traditional methods of pharmaceutical analysis, microfluidic chips are characterized by minimal sample demand and a significant improvement of detection sensitivity. The use of such chips may reduce consumption and costs significantly. Due to the characteristics of integration, miniaturization and automation, it is also known as ‘lab-on-a-chip’ (15). In the present study, the high throughput screening of the effective concentration of FK506 was determined using very small amounts of cells and drugs. The experimental conditions were easily controlled by changing the concentration of the FK506 solution, and the risk of contamination was minimal. More importantly, a stable culture environment was constituted and maintained by the continuous medium supply and waste removal system (16). Furthermore, FK506 diffused into the cell culture chamber, and the metabolic waste generated by cell growth was drained away simultaneously. This bionic design was used to test different concentrations of FK506 for their ability to improve SC growth. The design of parallel pipelines through from the side of the cell culture chamber kept cells away from the direct flushing effect of solution. Due to the low consumption of cells in microfluidic devices, cell loss can influence the result significantly. The small but effective design of the device in the present study may reduce the influence of cell loss. The consistency of the cell proliferation between the microfluidic chip and 96-well plates (Fig. 6) demonstrates that the microfluidic device performs excellently in the high throughput screening of effective drug concentrations. Due to the aforementioned advantages, microfluidic devices are ideal platforms for cell level manipulation and analysis *in vitro*.

FK506 is widely used clinically as an immunosuppressant following liver and kidney transplantations. By combining with FK506 binding protein-1 and suppressing the immune responses induced by nerve injury, it creates a suitable microenvironment for nerve regeneration (17,18). Although the effectiveness of FK506 for the promotion of nerve regeneration is known (19), at present, due to the fact that systemic application may lead to opportunistic infections and tumors and is not generally accepted for the treatment of peripheral nerve injury, FK506 has not been widely used for the treatment of simple peripheral nerve injury. The systemic application of FK506 has certain side-effects such as nephrotoxicity, gastrointestinal dysfunction and hypertension. These factors restrict the application of FK506. The method of local application of FK506 to avoid side-effects from systemic application has been widely approved by clinics (20). However, no accurate test is available to determine the effective concentration for local application. For the purpose of testing the effective concentration, a series of FK506 concentrations were established and their effects on rat SCs were investigated using the microfluidic device in the present study. It was shown that the cell proliferation rate increased as the FK506 concentration increased and reached a peak at 1.786±0.014 ng/ml. As the concentration of FK506 increased from 1.786±0.014 to 2.5±0.003 ng/ml, no further increase occurred, which indicates that higher concentrations have no additional promoting effect. Accordingly, it is considered that FK506 shows a maximum capacity for stimulating peripheral nerve regeneration at a concentration of 1.786±0.014 ng/ml. The risk of adverse effects is likely to be greatly increased at higher dosages.

In the present study, FK506 synergistically promoted SC proliferation. The effective FK506 concentration for local application was determined to be 1.786±0.014 ng/ml. A microfluidic device was fabricated from PDMS. The device design was developed in order to adapt to the cell culture and the effectiveness for high-throughput screening was demonstrated by the intensity of fluorescence of FITC-dextran. The data obtained revealed that the microfluidic device described in the present study is a candidate for cell level manipulation and analysis *in vitro*. The advantages of miniaturization and high integration indicate that microfluidic technology holds great promise for use in high-through screening at the cellular level.
